# The impact of socioeconomic status on outcomes in hepatocellular carcinoma: Inferences from primary insurance

**DOI:** 10.1002/cam4.2251

**Published:** 2019-08-22

**Authors:** Cortlandt M. Sellers, Johannes Uhlig, Johannes M. Ludwig, Tamar Taddei, Stacey M. Stein, Joseph K. Lim, Hyun S. Kim

**Affiliations:** ^1^ Section of Interventional Radiology, Department of Radiology and Biomedical Imaging Yale School of Medicine New Haven CT; ^2^ Department for Diagnostic and Interventional Radiology University Medical Center Goettingen Goettingen Germany; ^3^ Department of Diagnostic and Interventional Radiology and Neuroradiology University Hospital Essen, University of Duisburg‐Essen Essen Germany; ^4^ Section of Digestive Diseases, Department of Internal Medicine Yale School of Medicine New Haven Connecticut; ^5^ Section of Medical Oncology, Department of Internal Medicine Yale School of Medicine New Haven Connecticut; ^6^ Yale Cancer Center, Yale School of Medicine New Haven Connecticut

**Keywords:** cirrhosis, hepatitis C, hepatocellular carcinoma, insurance, socioeconomic status

## Abstract

**Background:**

To investigate the impact of insurance status on outcomes in patients with hepatocellular carcinoma (HCC).

**Methods:**

Patients diagnosed with HCC in the cancer registry from 2005 to 2016 were retrospectively stratified by insurance group. Overall survival was assessed via Kaplan‐Meier curves and Cox proportional hazard models including potential confounders in multivariable analyses.

**Results:**

Seven hundred and sixty‐nine patients met inclusion criteria (median age 63 years, 78.8% male, 65.9% Caucasian). 44.5% had private insurance (n = 342), 29.1% had Medicare (n = 224), and 26.4% had Medicaid (n = 203). At diagnosis, Medicaid patients had higher rates of Child‐Pugh B (32.0%) and C disease (23.6%) vs Medicare (28.6% and 9.8%) and private insurance (26.9% and 6.7%, *P* < 0.0001) and higher MELD scores (median 11.0) vs Medicare (9.0) and private insurance (9.0, *P* = 0.0266). Across insurance groups, patients had similar distribution of American Joint Committee on Cancer stage, tumor size, and multifocal tumor burden. Patients with private insurance had the highest survival (median OS 21.9 months) vs Medicare (17.7 months) and Medicaid (13.0 months, overall *P* = 0.0061). On univariate analysis, Medicaid patients demonstrated decreased survival vs private insurance (HR 1.40, 95% CI: 1.146‐1.715, *P* = 0.0011). After adjustment for liver disease factors, this survival difference lost statistical significance (Medicaid vs private insurance, HR 1.02, 95% CI: 0.819‐1.266, *P* = 0.8596).

**Conclusion:**

Medicaid was associated with advanced liver disease at HCC diagnosis; however, insurance status is not an independent predictor of HCC survival.

## Graphical abstract

Medicaid is associated with advanced liver cirrhosis at time of hepatocellular carcinoma diagnosis. Primary insurance is not an independent predictor of survival.

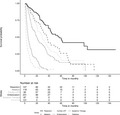

## 1 INTRODUCTION

Hepatocellular carcinoma (HCC) accounts for 70%‐85% of primary liver cancers; liver cancer is the fifth most common cancer worldwide and the third leading cause of cancer death.^1^, ^2^, ^3^ Approximately 60% of HCC cases are attributable to infection with hepatitis B (HBV) or hepatitis C (HCV) virus.^4^, ^5^, ^6^ Two other main causes of HCC are cirrhosis secondary to chronic alcohol consumption and nonalcoholic steatohepatitis (NASH). In the United States, rates of NASH‐driven HCC are increasing.^7^

The predominant curative therapies for HCC are liver resection or liver transplantation, although recent studies have reported that radiofrequency ablation may be as effective as resection in treating small solitary HCC lesions.^8^, ^9^, ^10^ Locoregional therapies (LRT), such as thermal ablation and transarterial chemoembolization, may be used as bridging therapy to transplant, to downstage disease, or as a palliative option. Treatment allocation is influenced by a variety of factors, including extent of cancer and the severity of liver disease. Patients with decompensated liver cirrhosis, poor hepatic synthetic function, and/or other serious comorbidities are less likely to receive treatment with curative intent. However, while patients with advanced cirrhosis are typically excluded from resection, these same patients are often prioritized for transplant.

Race, ethnicity, and socioeconomic status (SES) have been found to have significant effects on HCC incidence, overall survival, and treatment allocation.^11^, ^12^, ^13^, ^14^, ^15^, ^16^, ^17^ In the Swiss Hepatitis C Cohort study, low SES was associated with the development of HCC.^18^ Insurance status often correlates with SES. The purpose of our study was to examine the impact of primary insurance payer on outcomes in an inner‐city tertiary care hospital population, with the hypothesis that insurance would reflect SES and that patients with Medicaid coverage would have poorer survival compared to Medicare or private insurance.

## 2 MATERIALS AND METHODS

### 2.1 Study population

Institutional review board approval was obtained prior to the study. We identified adult patients from the Smilow Cancer Center cancer registry at Yale New Haven Hospital who were diagnosed with HCC via either radiologic or histopathologic criteria between 2005 and 2016 and followed through 2017. Patients with unknown treatment status or who received the majority of their treatment at another hospital were excluded. Patients with no or unknown insurance and patients with VA, Tricare, or Indian Health Service insurance were also excluded, as these groups were too small for statistical analysis. Patients who received liver transplantation and patients with combined HCC and cholangiocarcinoma were not included in this study.

### 2.2 Data collection

Data available from the registry included age, gender, ethnicity, primary insurance, American Joint Committee on Cancer (AJCC) staging, and treatment status. Further data, including baseline laboratory values, tumor imaging, and detailed treatment course, was acquired through electronic medical record review. Child‐Pugh score, Model of End‐stage Liver Disease (MELD) score, Barcelona Clinic Liver Cancer (BCLC) stage, and Charlson Comorbidity index (CCI) were calculated using baseline laboratory values, patient characteristics, and imaging reports.

### 2.3 Statistical analysis

Treatment was stratified into resection, ablation (percutaneous or laparoscopic), transcatheter LRT, combined ablation and transcatheter LRT (combo LRT), systemic chemotherapy, and palliative care. Therapy status and the temporal sequence of treatments received were explicitly identified via chart review. Patients receiving resection with or without additional therapies were categorized as “resection.” Patients receiving a type of LRT as well as chemotherapy or radiation therapy were classified under the type of LRT received. “Curative intent” treatment was defined as patients receiving either resection or ablation of a single lesion with a largest diameter of less than 3 cm.

The database explicitly identified primary insurance payer at the time of diagnosis, which was subsequently divided into three groups: private insurance, Medicare, and Medicaid. Patients with Medicare with supplemental insurance were classified as private insurance.

Categorical variables were compared using the χ^2^ test and continuous variables using the Kruskal‐Wallis test for nonnormally distributed data to identify key differences between insurance groups. The Kaplan‐Meier method was used to estimate median overall survival (OS). Prognostic factors for overall survival rates were compared via univariate (UVA) Cox proportional hazard models, including age, gender, ethnicity, Child‐Pugh class, MELD score, liver cancer etiology, BCLC stage, best AJCC stage, largest tumor diameter, tumor location, multifocal tumor burden, and treatment intent. Factors that were significant on UVA were included in the multivariable (MVA) proportional hazards analysis to account for confounding. An alpha level of 0.05 was chosen to indicate statistical significance. All *P*‐values provided are two‐sided. Calculations were performed using JMP Pro v.13.0.0 (SAS Institute Inc, Cary, NC) and R v.3.4.3 (R Core Development Team, Vienna, Austria).

## 3 RESULTS

### 3.1 Demographics

**Figure 1:**
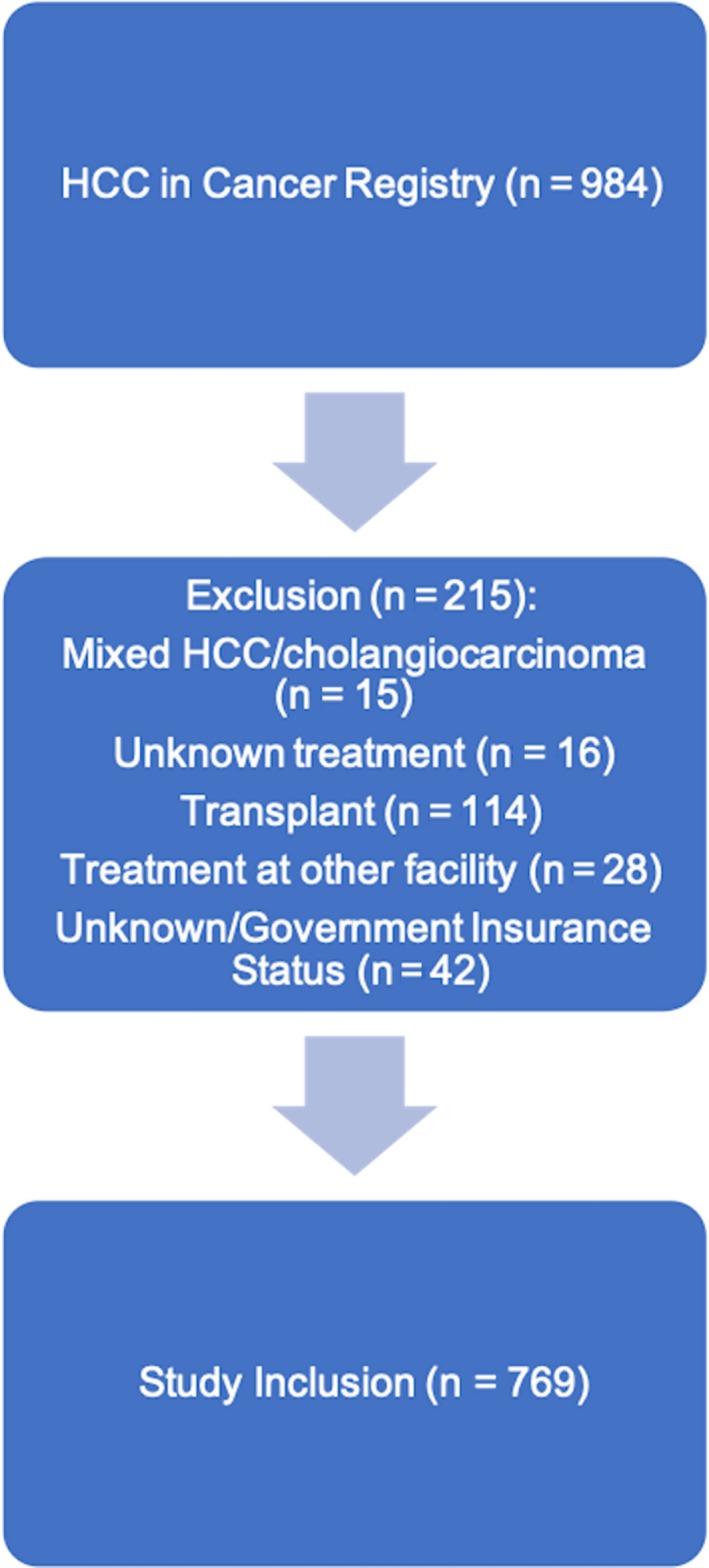
Flow chart demonstrating patient inclusion process

Of 984 potential participants, 769 met inclusion criteria (median age 63 years) (see Figure 1 for study design). Patients were 78.8% male (n = 606) and 65.9% Caucasian (n = 507). Four hundred and thirteen patients (53.7%) had hepatitis C‐related HCC, 35 patients (4.6%) had HBV‐related HCC, and 19 patients (2.5%) had combined HBV/HCV‐related HCC. Ninety‐nine patients (12.9%) had alcohol‐related HCC, 70 patients (9.1%) had NASH‐related HCC, and 133 patients (17.3%) had HCC from other etiologies, including autoimmune cirrhosis, Wilson's disease, hemochromatosis, and unknown etiology. Four hundred and twenty‐six patients had Child‐Pugh A disease (55.4%), 221 patients had Child‐Pugh B disease (28.7%), and 93 patients had Child‐Pugh C disease (12.1%). Two hundred and sixty‐six patients had very early or early BCLC stage disease (34.6%). 44.5% of patients had private insurance (n = 342), 29.1% had Medicare (n = 224), and 26.4% had Medicaid (n = 203). One hundred and seven patients (13.9%) underwent resection, 118 (15.3%) received thermal ablation, 231 had transcatheter LRT (30.0%), 95 underwent combo LRT (12.4%), 87 received systemic therapy (11.3%), and 131 received palliative care (17.0%). The rate of curative intent treatment for the entire cohort was 24.3% (187 pts). Median follow‐up time was 13.9 months, with the primary end point being death or the end of the study (0.03 months – 150.4 months). See Table 1 for further baseline demographic data. The proportion of missing data was ≤10.0% for all variables.

**Table 1:** Patient characteristics by insurance status

Factor	Total (n = 769)	Medicaid (n = 203)	Medicare (n = 224)	Private (n = 342)	*P*‐value
Age					<0.0001
Median (Mean ± SD)	63.0 (63.6 ± 10.3)	57.0 (57.3 ± 7.5)	68.0 (69.1±8.9)	63.0 (63.8 ± 10.4)	
Male gender	606 (78.8%)	168 (82.8%)	169 (75.5%)	269 (78.6%)	0.1773
Race/ethnicity					<0.0001
Caucasian	507 (65.9%)	103 (50.7%)	151 (67.4%)	253 (74.0%)	
African American	106 (13.8%)	40 (19.7%)	30 (13.4%)	36 (10.5%)	
Hispanic	118 (15.3%)	52 (25.6%)	33 (14.7%)	33 (9.7%)	
Other/unknown	38 (4.9%)	8 (3.9%)	10 (4.5%)	20 (5.9%)	
Comorbidities (CCI)					<0.0001
Median (Mean ± SD)	7.0 (7.2 ± 2.4)	6.0 (6.7 ± 2.1)	8.0 (8.0± 2.5)	7.0 (7.0± 2.3)	
HCC etiology					<0.0001
HCV	413 (53.7%)	148 (72.9%)	107 (47.8%)	158 (46.2%)	
HBV	35 (4.6%)	7 (3.5%)	6 (2.7%)	22 (6.4%)	
HBV/HCV	19 (2.5%)	10 (4.9%)	5 (2.2%)	4 (1.2%)	
Ethanol	99 (12.9%)	18 (8.9%)	24 (10.7%)	57 (16.7%)	
NASH	70 (9.1%)	8 (3.9%)	29 (13.0%)	33 (9.7%)	
Other	133 (17.3%)	12 (5.9%)	53 (23.7%)	68 (19.9%)	
Child‐Pugh score				<0.0001	
A	426 (55.4%)	81 (39.9%)	132 (58.9%)	213 (62.3%)	
B	221 (28.7%)	65 (32.0%)	64 (28.6%)	92 (26.9%)	
C	93 (12.1%)	48 (23.6%)	22 (9.8%)	23 (6.7%)	
Missing	29 (3.8%)	9 (4.4%)	6 (2.7%)	14 (4.1%)	
MELD					0.0002
Median (Mean ± SD)	9.0 (10.9 ± 5.0)	11.0 (12.1 ± 5.5)	9.0 (10.7 ± 4.9)	9.0 (10.4 ± 4.7)	
Missing	11 (1.4%)	3 (1.4%)	4 (1.8%)	4 (1.2%)	
MELD‐Na					0.0002
Median (Mean ± SD)	10.0 (11.5 ± 5.7)	11.0 (12.9 ± 6.2)	9.0 (11.1 ± 5.5)	9.0 (10.9 ± 5.4)	
Missing	12 (1.6%)	4 (2.0%)	4 (1.8%)	4 (1.2%)	
BCLC stage					0.0210
0 or A	266 (34.6%)	59 (29.1%)	87 (38.8%)	120 (35.1%)	
B	105 (13.7%)	24 (11.8%)	33 (14.7%)	48 (14.0%)	
C	331 (43.0%)	91 (44.8%)	87 (38.8%)	153 (44.7%)	
D	64 (8.3%)	28 (13.8%)	17 (7.6%)	19 (5.6%)	
Missing	3 (0.4%)	1 (0.5%)	0 (0%)	2 (0.6%)	

Factor	Total (n = 772)	Medicaid (n = 203)	Medicare (n = 320)	Private (n = 249)	*P*‐value
AJCC stage					0.9191
1	317 (41.2%)	82 (40.4%)	101 (45.1%)	134 (39.2%)	
2	173 (22.5%)	49 (24.1%)	45 (20.1%)	79 (23.1%)	
3	143 (18.6%)	37 (18.2%)	43 (19.2%)	63 (18.4%)	
4	96 (12.5%)	27 (13.3%)	28 (12.5%)	41 (11.9%)	
Missing	40 (5.2%)	8 (3.9%)	7 (3.1%)	25 (7.3%)	
Tumor size					0.2422
Median (Mean ± SD)	3.4 (4.8 ± 3.8)	3.2 (4.5 ± 3.7)	3.4 (4.6 ± 3.5)	3.6 (5.1 ± 4.0)	
Missing	32 (4.2%)	10 (4.9%)	12 (5.4%)	10 (2.9%)	
Tumor location				0.0014	
Right	353 (45.9%)	101 (49.8%)	94 (42.0%)	158 (46.2%)	
Left	120 (15.6%)	26 (12.8%)	54 (24.1%)	40 (11.7%)	
Bilobar	276 (35.9%)	73 (36.0%)	69 (30.8%)	134 (39.2%)	
Missing	20 (2.6%)	3 (1.5%)	7 (3.1%)	10 (2.9%)	
Multifocal tumor burden					0.4317
Yes	358 (46.6%)	102 (50.2%)	100 (44.6%)	156 (45.6%)	
No	406 (52.8%)	99 (48.8%)	122 (54.5%)	185 (54.1%)	
Missing	5 (0.7%)	2 (1.0%)	2 (0.9%)	1 (0.3%)	
AFP					0.0077
Median (Mean ± SD)	25.0 (10398.5 ± 62212.2)	43.0 (10556.8 ± 43863.2)	14.5 (10005.2 ± 81859.0)	24.0 (10561.3 ± 77103.3)	
Missing	19 (2.5%)	4 (2.0%)	6 (2.7%)	9 (2.6%)	
Treatment Group					0.0024
Resection	107 (13.9%)	13 (6.4%)	33 (14.7%)	61 (17.8%)	
Ablation	118 (15.3%)	33 (16.3%)	31 (13.8%)	54 (15.8%)	
Transcatheter LRT	231 30.0%)	57 (28.1%)	65 (29.0%)	109 (31.9%)	
Combo LRT	95 (12.4%)	27 (13.3%)	30 (13.4%)	38 (11.1%)	
Systemic therapy	87 (11.3%)	24 (11.8%)	24 (10.7%)	39 (11.4%)	
alliative	131 (17.0%)	49 (24.1%)	41 (18.3%)	41 (12.0%)	

Abbreviations used: SD, standard deviation; BMI, body mass index; CI, confidence interval; HCC, hepatocellular carcinoma; HCV, hepatitis C virus; HBV, hepatitis B virus; NASH, nonalcoholic steatohepatitis; MELD, Model for End‐Stage Liver Disease; MELD‐Na, MELD including sodium; BCLC, Barcelona Clinic Liver Cancer staging system; AJCC, American Joint Committee on Cancer staging system; AFP, alpha‐fetoprotein; LRT, locoregional therapy.

### 3.2 Overall survival

**Figure 2:**
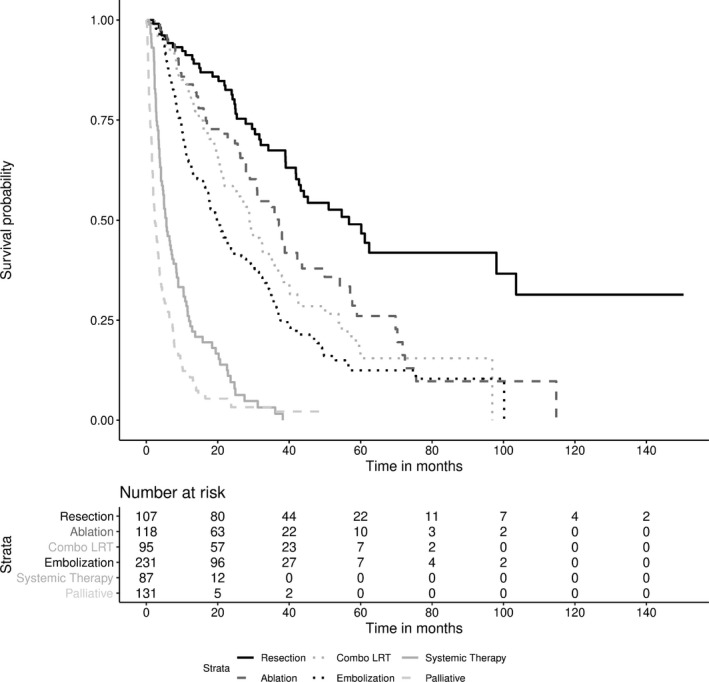
Survival curves of HCC by treatment group

Median overall survival from diagnosis of the cohort was 19.2 months. 1‐, 3‐, and 5‐year survivals were 59.6%, 31.2%, and 16.9%, respectively. Patients who received resection had the highest survival (median OS 56.7 months), followed by ablation (37.1 months), combo LRT (28.9 months), transcatheter LRT (19.8 months), systemic therapy (5.6 months), and palliative care (2.4 months, overall *P* < 0.0001) as presented in Figure 2. The curative intent treatment group demonstrated increased survival (median OS 43.6 months) vs the noncurative intent treatment group (12.5 months, *P* < 0.0001).

### 3.3 Prognostic factors in HCC

Prognostic factors for HCC associated with decreased survival on UVA included increased comorbidities (HR 1.2, 95% CI:1.14‐1.22, *P* < 0.0001); Child‐Pugh class B or C disease (B vs A, HR 2.3, 95% CI:1.86‐2.73, *P* < 0.0001; C vs A HR 3.6, 95% CI:2.75‐4.55, *P* < 0.0001); increased MELD score (HR 1.1, 95% CI: 1.06‐1.09, *P* < 0.0001); BCLC stage B (B vs 0/A, HR 2.4, 95% CI:1.84‐3.23, *P* < 0.0001), C (C vs 0/A, HR 2.8, 95% CI: 2.31‐3.53, *P* < 0.0001), or D (D vs 0/A, HR 4.1, 95% CI: 2.99‐5.61, *P* < 0.001); AJCC stage 2 (2 vs 1; HR 1.4, 95% CI: 1.10‐1.75, *P* = 0.0064), 3 (3 vs 1; HR 2.8, 95% CI: 2.24‐3.57, *P* < 0.0001) or 4 (4 vs 1; HR 6.0, 95% CI: 4.57‐7.75, *P* < 0.0001); increased tumor size (HR 1.1, 95% CI:1.05‐1.09, *P* < 0.0001); bilobar tumors (HR 2.0, 95% CI: 1.67‐2.42, *P* < 0.0001); multifocal tumor burden (HR 2.0, 95% CI:1.69‐2.39, *P* < 0.0001); and log‐transformed AFP (HR 1.2, 95% CI: 1.14‐1.20, *P* < 0.0001). Treatment status was also a significant prognostic factor on UVA (see Table 2 for more detail).

**Table 2:** Significant prognostic factors in HCC on univariate analysis

Factor	HR	lower 95%	upper 95%	*P*‐value
Comorbidities (continuous)	1.18	1.144	1.220	<0.0001
Insurance Status				
Private (reference)				
Medicare	1.13	0.915	1.383	0.2590
Medicaid	1.40	1.146	1.715	0.0011
Child Pugh score				
A (reference)				
B	2.26	1.860	2.730	<0.0001
C	3.56	2.750	4.553	<0.0001
MELD	1.07	1.059	1.089	<0.0001
BCLC				
0 or A (reference)				
B	2.44	1.838	3.230	<0.0001
C	2.85	2.313	3.528	<0.0001
D	4.12	2.991	5.606	<0.0001
AJCC stage				
1 (reference)				
2	1.39	1.098	1.753	0.0064
3	2.84	2.245	3.574	<0.0001
4	5.97	4.568	7.752	<0.0001
Tumor size (cm)	1.07	1.051	1.091	<0.0001
Tumor location				
Right (reference)				
Left	0.98	0.750	1.268	0.8844
Bilobar	2.01	1.667	2.418	<0.0001
Multifocal tumor burden	2.01	1.693	2.386	<0.0001
Log(AFP)	1.17	1.138	1.204	<0.0001
Treatment				
Resection (reference)				
Ablation	1.77	1.210	2.599	<0.0001
Transcatheter LRT	3.04	2.176	4.260	<0.0001
Combo LRT	2.26	1.539	3.304	<0.0001
Systemic therapy	10.05	6.868	14.697	<0.0001
Palliative	17.86	12.466	25.596	<0.0001

Abbreviations: HCC, hepatocellular carcinoma; HR, hazard ratio; MELD, Model for End‐Stage Liver Disease; BCLC, Barcelona Clinic Liver Cancer staging system; AJCC, American Joint Committee on Cancer staging system; cm, centimeters; AFP, alpha‐fetoprotein; LRT, locoregional therapy.

On multivariable proportional hazards analysis, factors associated with decreased survival included increased comorbidities (HR 1.08, 95% CI: 1.04‐1.12, *P* = 0.0002); Child‐Pugh B disease (B vs A, HR 1.61, 95% CI: 1.26‐2.06, *P* = 0.0001); increased MELD score (HR 1.03, 95% CI: 1.003‐1.06, *P* = 0.0264); BCLC stage B (B vs 0/A; HR 1.61, 95% CI: 1.13‐2.29, *P* = 0.0081), C (C vs 0/A, HR 1.51, 95% CI: 1.14‐2.00, *P* = 0.0043), or D (D vs 0/A; HR 2.16, 95% CI: 1.39‐3.37, *P* = 0.0006); AJCC stage 3 (3 vs 1; HR 1.75, 95% CI: 1.25‐2.45, *P* = 0.0011) or 4 disease (4 vs 1; HR 1.89, 95% CI: 1.23‐2.89, *P* = 0.0040); tumor size (HR 1.04, 95% CI: 1.01‐1.07, *P* = 0.0217); bilobar tumor burden (vs right‐sided tumors; HR 1.38, 95% CI: 1.07‐2.77, *P* = 0.0127); log‐transformed AFP (HR 1.05, 95% CI: 1.01‐1.09, *P* = 0.0075), and treatment status. (see Table 3).

**Table 3:** Significant prognostic factors in HCC on multivariable analysis

Factor	HR	lower 95%	upper 95%	*P*‐value
Comorbidities (continuous)	1.08	1.036	1.122	0.0002
Child Pugh score				
A (reference)				
B	1.61	1.264	2.059	0.0001
MELD (continuous)	1.03	1.004	1.060	0.0264
BCLC				
0/A (reference)				
B	1.61	1.132	2.293	0.0081
C	1.51	1.137	1.996	0.0043
D	2.16	1.393	3.357	0.0006
AJCC stage				
1 (reference)				
3	1.75	1.252	2.455	0.0011
4	1.89	1.226	2.890	0.0040
Tumor size (continuous)	1.04	1.005	1.067	0.0217
Tumor location				
Right (reference)				
Bilobar	1.38	1.071	1.770	0.0127
Log(AFP)	1.05	1.013	1.086	0.0075
Treatment				
Resection (reference)				
Ablation	1.39	0.881	2.191	0.1566
Transcatheter LRT	1.81	1.247	2.640	0.0019
Combo LRT	2.07	1.332	3.218	0.0012
Systemic therapy	4.04	2.536	6.429	<0.0001
Palliative	7.70	4.875	12.152	<0.0001

Abbreviations: HCC, hepatocellular carcinoma; HR, hazard ratio; BCLC, Barcelona Clinic Liver Cancer staging system; AJCC, American Joint Committee on Cancer staging system; AFP, alpha‐fetoprotein; LRT, locoregional therapy.

### 3.4 Insurance status and overall survival

**Figure 3:**
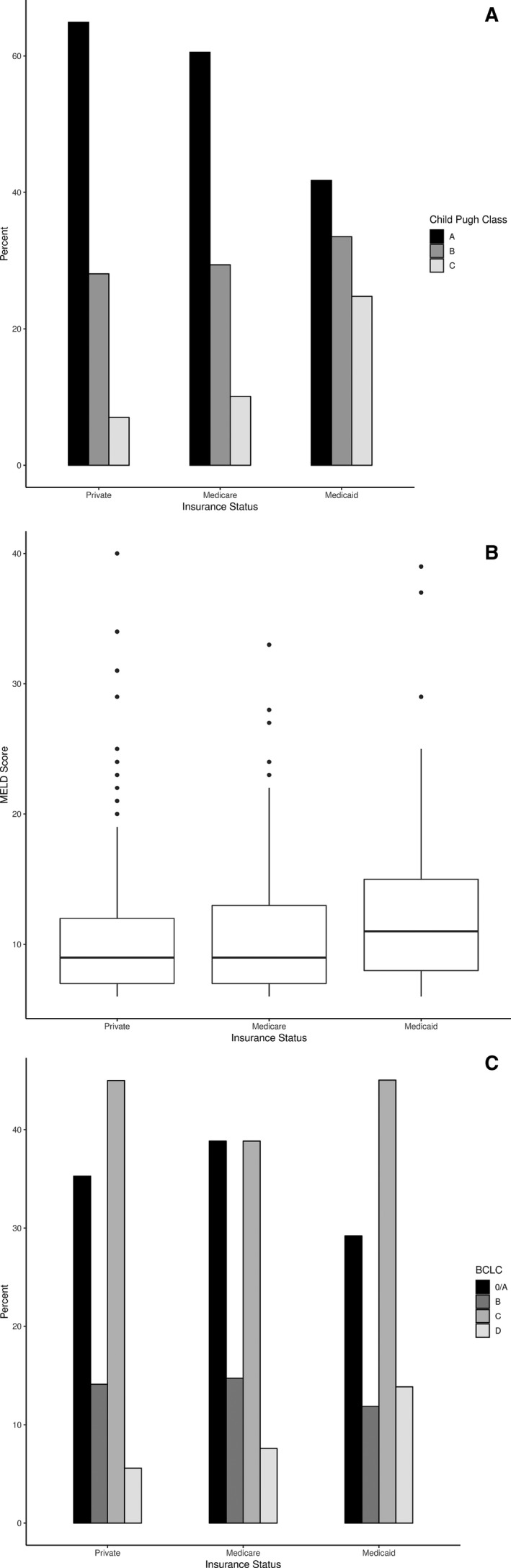
(A) Distribution of Child‐Pugh class by insurance group. (B) Distribution of MELD score by insurance group. (C) Distribution of BCLC stage by insurance group

Medicare patients were older (median age 68 years) than Medicaid patients (57 years) or private insurance patients (63 years, overall *P* < 0.0001) and had more comorbidities (median CCI = 8.0) vs Medicaid (6.0) or private insurance (7.0, overall *P* < 0.0001). Medicaid patients were more likely to be African American (19.7%) or Hispanic (25.6%) than Medicare patients (13.4% and 14.7% respectively) or private insurance patients (10.5% and 9.7%, overall *P* < 0.0001). Higher rates of viral hepatitis‐related HCC were seen in Medicaid patients (81.3%) vs Medicare (52.7%) or private insurance (53.8%, overall *P* < 0.0001). Patients with private insurance had the highest rates of alcohol‐related HCC (16.7%) followed by Medicare (10.7%) and Medicaid (8.9%, *P* < 0.0001). 13.0% of Medicare patients had NASH‐related HCC vs private (9.7%) vs Medicaid (3.9%, *P* < 0.0001). Medicaid patients demonstrated higher rates of Child‐Pugh B (32.0%) and C disease (23.6%) vs Medicare (28.6% and 9.8%, respectively) and private insurance (26.9% and 6.7%, overall *P* < 0.0001) (see Figure 3A) as well as higher median MELD score (11.0) vs Medicare (9.0) and private (9.0, overall *P* = 0.0002) (Figure 3B). Medicaid patients had lower rates of BCLC stage 0/A (29.1%) than Medicare (38.8%) and private (35.1%) and higher rates of BCLC stage D disease (13.8%) vs Medicare (7.6%) and private (5.6%) (overall *P* = 0.0210) (Figure 3C).

Medicaid patients had higher median AFP (43.0) vs Medicare (14.5) or private insurance (24.0, overall *P* = 0.0077). Patients with Medicare had a higher percentage of left‐sided tumors (24.1%) vs Medicaid (12.8%) vs private insurance (11.7%, overall *P* = 0.0014). However, patients were similar in terms of AJCC stage, tumor size, and the presence of multifocal tumors across insurance groups. Those with Medicaid were less likely to receive resection (6.4%) than Medicare patients (14.7%) or private insurance patients (17.8%) and more likely to receive palliative care (24.1% vs 18.3% vs 12.0% respectively, overall *P* = 0.0024). Curative intent treatment was highest in private insurance patients (28.1%) vs Medicare (24.1%) vs Medicaid (18.2%, *P* = 0.0317). However, when stratified by Child‐Pugh class, there were no significant differences in treatment allocation between insurance groups.

**Figure 4:**
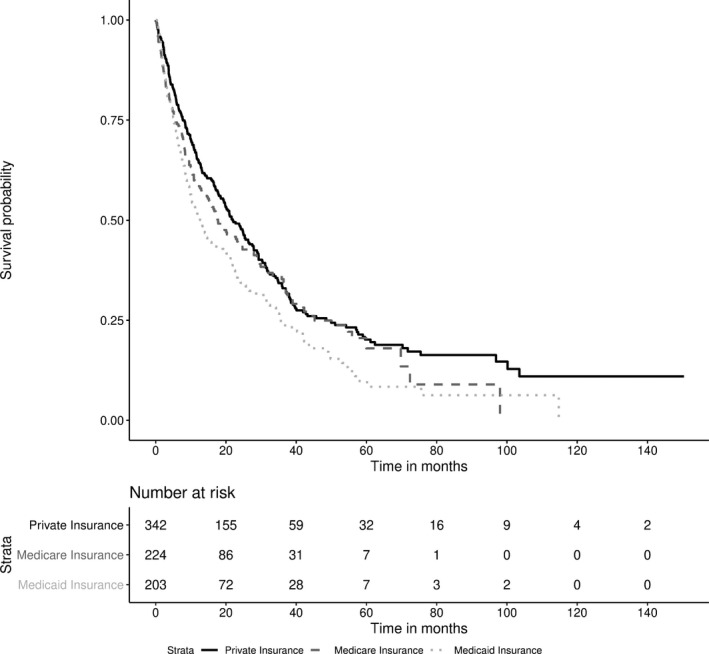
Survival curves of HCC by primary insurance status

Private insurance patients had the highest median overall survival (median OS 21.9 months) vs Medicare (17.7 months), and Medicaid (13.0 months, overall *P* = 0.0061) (see Figure 4). On UVA, the Medicaid group had decreased survival vs private insurance (HR 1.40, 95% CI: 1.146‐1.715, *P* = 0.0011). There was a trend towards poorer survival in Medicaid patients vs Medicare patients (HR 1.25, 95% CI: 0.996‐1.560, *P* = 0.0544), but this did not reach significance. After incorporating Child‐Pugh score and MELD score into a multivariable model, the survival differences between Medicaid and private insurance lost statistical significance (Medicaid vs private, HR 1.02, 95% CI: 0.819‐1.266, *P* = 0.8596).

## 4 DISCUSSION

### 4.1 Disease presentation

In our study, patients with Medicaid presented with more advanced liver disease as represented by Child‐Pugh and MELD score than patients with either Medicare or private insurance. However, no significant differences between insurance groups existed for cancer stage, as measured by AJCC stage, tumor size, tumor location, and the presence of multifocal HCC. While BCLC stage differed across insurance groups, this is likely due to BCLC including liver disease and performance status in the staging algorithm, while AJCC staging does not.^19^, ^20^, ^21^ It has been noted that HCC patients with Medicaid or no insurance are more likely to present with late‐stage cancer vs patients with private insurance.^22^, ^23^ Lack of long‐term insurance has also been associated with higher prevalence of metastases in both the University Health Consortium [OR 1.9, 95% CI: 1.6‐2.2] and Nationwide Inpatient Small [OR 1.6, 95% CI: 1.4‐1.9] databases.^24^

### 4.2 Treatment allocation

Curative intent treatment was defined in this study as partial hepatic resection or ablation of a single lesion <3 cm in diameter and occurred less frequently in Medicaid patients than in those with Medicare or private insurance. Furthermore, the highest rates of palliative care occurred in Medicaid patients. These differences in treatment allocation disappeared after substratifying insurance groups by Child‐Pugh score. Hence, it can be inferred that treatment differences were due to increased prevalence of severe liver disease in the Medicaid group. This additionally demonstrates that treatment decisions at this institution are independent from insurance status. Primary insurance can serve as a surrogate parameter for SES. Various studies have reported that patients with Medicaid, underinsurance, no insurance, or lower SES are less likely to receive surgical treatment^25^, ^26^ or even treatment in general.^23^, ^27^, ^28^ After controlling for tumor stage, resection status, and transplant eligibility, Sarpel found that patients with government insurance (Medicaid or Medicare without supplement) were less likely to undergo transplantation for HCC.^17^ Similarly, in a study of safety‐net hospitals, vulnerable patients (including those with Medicaid and poor SES) had lower rates of curative surgery and poorer short‐term outcomes.^29^ However, safety‐net patients who could endure liver surgery had a similar prognosis as compared with patients at nonsafety net hospitals. This further suggests that survival differences between socioeconomic and insurance groups may be driven more by liver and tumor factors than by treatment decisions.

### 4.3 Overall survival and insurance status

Kaplan‐Meier curves and univariate Cox models demonstrated decreased survival in the Medicaid group as compared to the Medicare and private insurance groups in our cohort, which agreed with our original hypothesis. After analyzing the UNOS (United Network for Organ Sharing) database from 2002 to 2013, Magnetta reported that private payer insurance led to improved overall survival after OLT as compared to public insurance (Medicare/Medicaid) (MVA HR 0.91, 95% CI: 0.88‐0.93, *P* < 0.001).^30^ Hoehn similarly noted improved survival with private vs nonprivate insurance on a study of National Cancer Database patients with curable HCC (stage I/II) from 1998 to 2011.^14^ Poorer survival has also been associated with lower SES.^11^, ^12^, ^16^ In a study examining HCC survival over three decades, Wang et al found inferior survival in the high‐poverty group.^31^ In addition, Major has reported higher chronic liver disease mortality in areas of socioeconomic deprivation (HR 1.78, 95% CI: 1.34‐2.36).^32^

Although UVA showed decreased survival in Medicaid patients, primary insurance was not an independent predictor of survival after adjusting for Child‐Pugh and MELD scores. This is similar to what was described by Yu et al: while their results also showed that insurance was not an independent predictor of mortality in HCC, it was associated with more advanced liver disease at diagnosis and lower rates of transplantation.^33^ The lack of survival differences in our cohort after adjusting for liver disease factors demonstrates that patients with similar liver and performance status received similar treatments at this institution, regardless of insurance or SES. This suggests that while patients with lower SES or less desirable insurance may present with more advanced liver disease or cancer, if patients receive the appropriate treatment for their HCC stage and underlying liver disease, insurance and perhaps even SES may not affect survival.

### 4.4 Limitations

While this study relied on archived records, we limited the study to patients with complete insurance and treatment data. The degree of missing data in other patient characteristic variables was minimal and was distributed throughout insurance groups. Although our sample size may have led to underpowering of analyses in subgroups, this cohort is among the largest from a single institution to analyze the impact of insurance on survival in HCC. Finally, this study was conducted using a Western (US) population, and therefore results may not be globally generalizable.

## 5 CONCLUSION

Insurance status is not an independent predictor of HCC survival. Medicaid was associated with advanced liver disease at HCC diagnosis. After adjusting for Child‐Pugh score, rates of treatment with curative intent were similar between insurance groups.

## CONFLICT OF INTEREST

All authors state that they have no conflict of interest. The authors certify that they have no affiliations with or involvement in any organization or entity with any financial interest in the subject matter or materials discussed in this manuscript. Hyun S. Kim served on Advisory boards for Boston Scientific and SIRTex.

## AUTHOR CONTRIBUTIONS

CMS and HSK were involved in conceptualization, validation, and resources. CMS, JU, and JML were involved in formal analysis. CMS, JU, JML, and HSK were involved in methodology. CMS, JU, JML, and HSK were involved in investigation and data curation. CMS, JU, JML, TT, SMS, JKL, and HSK were involved in writing—original draft, edit, and final approval. CMS was involved in visualization. HSK was involved in supervision and project administration.

## Data Availability

Data available on request to the corresponding author due to privacy/ethical restrictions.
